# The impact of global health outreach experiences on medical student burnout

**DOI:** 10.1186/s12909-023-04451-6

**Published:** 2023-06-26

**Authors:** Thomas Kuehn, Cody Crandall, Jefferson Schmidt, Zeke Richards, Taylor Park, Morgan Szczepaniak, Isain Zapata, Mark Wardle

**Affiliations:** 1 Department of Primary Care, Rocky Vista University College of Osteopathic Medicine, 255 E. Center Street, Ivins, UT 84738 USA; 2 Department of Biomedical Sciences, Rocky Vista University College of Osteopathic Medicine, Parker, CO 80112 USA

**Keywords:** Medical Student, Burnout, CBI, Global Health Outreach Experience, Mission trip

## Abstract

**Background:**

Student burnout during medical education is a prevalent and critical problem. Burnout has reaching consequences, including negative health outcomes for students, financial loss for schools, and worsened patient care as students transition to practice. Global Health Outreach Experiences (GHOEs), known to enhance cultural awareness and clinical knowledge among medical students, are offered in most programs. Prior studies document that GHOEs benefit physicians suffering from burnout, with effects demonstrating improvement over 6 months. No study, to our knowledge, has assessed the influence GHOEs may have on medical student burnout with a comparable control group. This study examines whether participation in a GHOE, compared to a standard break from school, has a positive effect on burnout.

**Methods:**

A case control study utilizing the Copenhagen Burnout Inventory was conducted on medical students. 41 students participated in a one-week, spring break GHOE and 252 were randomly selected as non-participating students in a control group. Assessments were gathered 1 week prior, 1 week after, and 10 weeks after spring break. Response across the surveys in chronological order included 22, 20, 19 GHOE and 70, 66, 50 control participants.

**Results:**

A significant reduction in personal burnout (PB) (P = 0.0161), studies related burnout (SRB) (P = 0.0056), and colleagues related burnout (CRB) (P = 0.0357) was found among GHOE attendees compared to control participants at 10-weeks after spring break. When modeled with potential confounders, CRB and SRB reductions remained significant.

**Conclusion:**

GHOEs may be a potential tool for institutions to combat burnout rates in their students. The benefits of GHOEs appear to enhance over time.

**Supplementary Information:**

The online version contains supplementary material available at 10.1186/s12909-023-04451-6.

## Background

Alarming rates of burnout have been identified amongst medical students. Current studies suggest that at least half of all medical students experience burnout [1–3], with increasing occurrence over the 4-year curriculum [4, 5]. Burnout in medical students may have significant impacts, leading to a decline in mental health manifested by suicidal ideation [6–8], substance abuse [9], and depression [10]. Other consequences may include decreased empathy [11, 12], worsening professional conduct [12] and financial loss for schools through student dropout [12, 13]. The presence of burnout in students persists during transition into clinical training and residency, and can ultimately lead to suboptimal patient care through increased medical errors, and decreased proficiency [6, 14, 15].

Burnout was first characterized as emotional and physical exhaustion due to stressors at work [16]. The definition has since expanded to include depersonalization and low sense of accomplishment due to any emotionally demanding experience [17, 18]. It has been demonstrated that the workplace and not personal traits lead to burnout [1, 19]. Medical school curriculum and learning environment are thought to be main contributors to burnout in medical students [6].

Multiple institutional changes already exist to combat medical student burnout [6]. Student wellness programs and 24/7 access to mental health resources are required for accreditation [20, 21]. Didactic grading and board scoring have transitioned to pass/fail [22]. However, given the persistence of burnout as reported in recent literature, we are led to believe such institutional changes are insufficient [23, 2]. A multifaceted approach has been suggested to remediate burnout [23, 6], and thus, we explored a more individual-level intervention.

Relatively little research exists concerning the effectiveness that global health outreach experiences (GHOEs) have on addressing burnout in medical students. GHOEs, sometimes referred to as medical mission trips, or short-term experiences in global health, have been shown to improve cultural awareness, enhance clinical skills and knowledge of medical students [24–28]. The effects of GHOEs on medical student burnout are worth investigating for several reasons. GHOEs are a staple amongst the extracurricular opportunities medical schools offer. It has been reported that almost two-thirds of medical students anticipate such an experience to be part of their medical education [29]. Many students returning from such experiences claim a renewed sense of purpose, gratitude, connection, and drive at work [30, 31].

Current literature shows that short-term GHOEs may “reinvigorate and reengage physicians on the verge of or suffering from persistent burnout syndrome” [29]). For example, Campbell et al. saw improvement in burnout for physicians and nurses who participated in a short-term medical mission trip shortly after the experience with greater improvements at the 6-month follow-up [24]. Yet, no studies to our knowledge exist which assess the effects of GHOEs on medical student burnout specifically. A 2020 study involving pediatric and internal medicine residents saw that trainees who participated in GHOEs reported higher empathy, though no association between burnout reduction and GHOE involvement was noted [32]. It appears that GHOE involvement may not affect physicians and trainees equally, but with very few studies, it is difficult to conclude. Thus, we attempted to contribute to this gap in the literature by assessing the possible influence that GHOEs may have on medical student burnout.

With this project, we aimed to evaluate the effect of short-term GHOEs on the levels of burnout in first- and second-year medical students. Recognizing the positive effects that GHOEs have on physician burnout, we hypothesized that involvement in GHOEs during pre-clinical medical education could serve as a safeguard against burnout syndrome in medical students.

## Methods

### Participants, assessment schedule, and global trip description

**Fig. 1:**
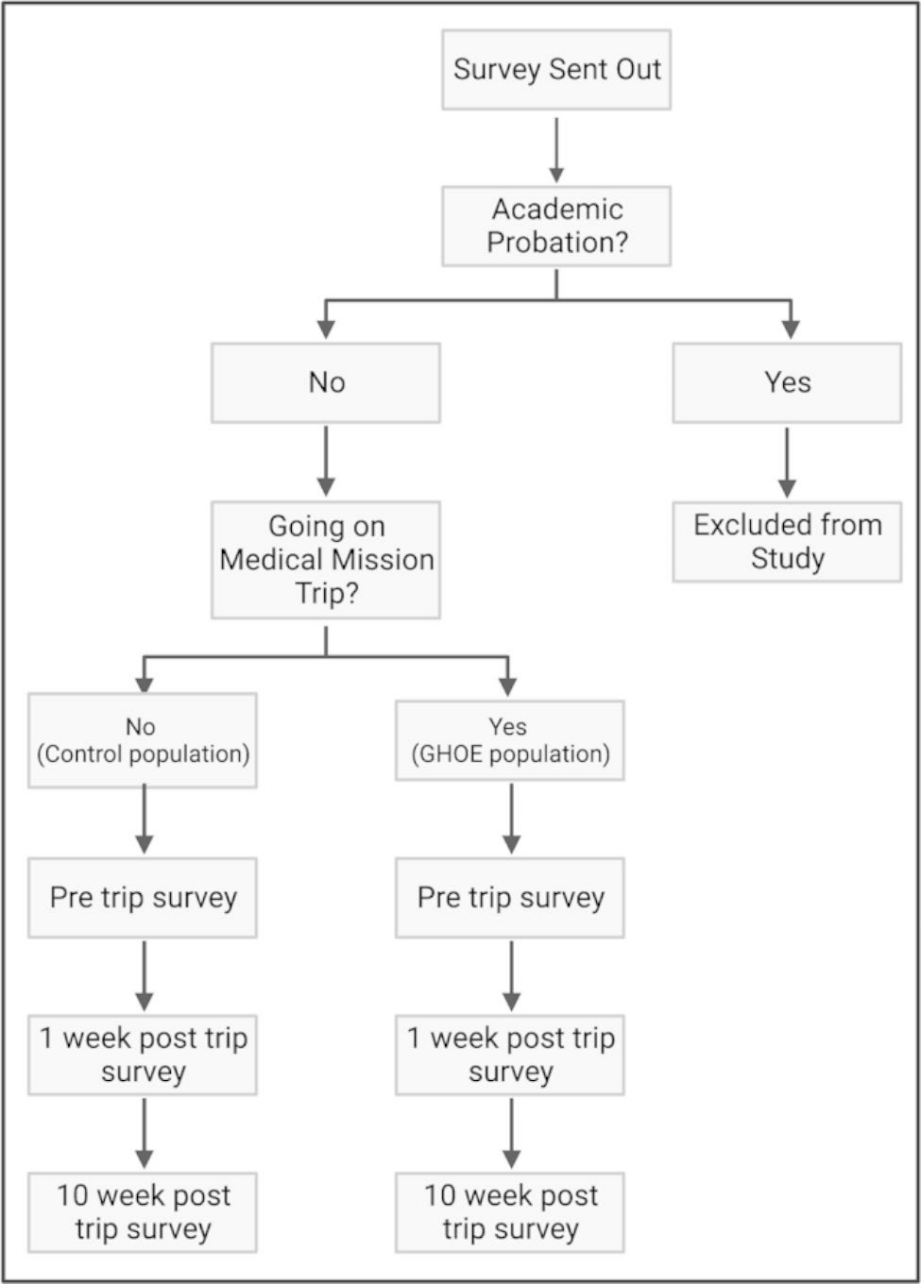
“Distribution of Survey” This flowchart depicts the distribution the survey, including initial exclusion criteria and separation into GHOE group and control group

An online survey was created using Qualtrics and administered via email to students at Rocky Vista University (RVU). The survey distribution is depicted in Fig. 1. 41 students, both OMS (Osteopathic Medical Student) I and OMS II attending either of two RVU sponsored global medicine trips over spring break, were selected and invited to respond to the survey. 252 OMS I and OMS II students who were not attending a global medicine trip at RVU were randomly selected using a random number generator from a pool of all OMS I and OMS II students at RVU. This group served as a control to compare with trip participant responses. Informed consent was obtained from all subjects prior to completion of each survey. Research group was designated in response to the question “Are you planning to attend an RVU Spring Break Global Trip in 2022? “. Students were excluded from the study if they were currently on academic probation. All participants consented to participate in this study in a voluntary manner with the option to withdraw at any time. This study was vetted by the RVU Institutional Review Board (IRB #: 2020-0014).

An identical survey was followed up 2 additional times over a 4-month period to GHOE participants, and the control group. Each survey was available for one week after distribution. The timing of the surveys is below:

Survey 1: 1 week before spring break to establish a baseline, February 2022.

Survey 2: 1 week after spring break to assess short-term effects, March 2022.

Survey 3: 10 weeks after spring break to assess long-term effects, May 2022.

The 3 surveys were able to be linked by the students creating a consistent unique code that they entered on each survey. These links were asked to be created in a way that kept the surveys anonymous to researchers in the study yet would allow for analysis of individual change of burnout over time.

Demographics collected on all survey participants included class, campus (UT or CO), gender, and age. All participants were asked to identify religiousness/spirituality, hours of leisure activity per week, and prior participation in GHOEs. GHOE participants were additionally asked to rank motivation for attendance to qualitatively assess GHOE participant intentions. Non-participants were asked to select their alternative spring break activities among the options of reset/relaxation, travel, clinical experience, service, and studying. This data was used to qualitatively evaluate the control group activities as an appropriate comparison.

No structure or guidance was given to the non-trip participants on how to use their time during spring break, but their planned activities (survey 1) and then completed activities (surveys 2 & 3) were assessed. The structure of the 2 GHOEs were similar. The two outreach trips went to remote areas of Ecuador and the Dominican Republic. Both trips required the use of local interpreters. Participants of both trips attended approximately 2 h of preparatory training spread out over 3 sessions that covered topics such as logistics, packing, culture and customs, team dynamics, personal health, and trip safety. Trip length for both locations was 8 days including an arrival day, an orientation day, 4 clinical days, 1 cultural adventure day, and a departure day. Clinical days consisted of the whole group traveling together to remote villages, setting up the clinical area, seeing patients in groups of 2–3 students under the supervision of physicians for 6–8 h, breaking down the clinic, and then traveling back to the hotel. The cultural adventure day was a day of rest, relaxation, and guided exploration of the local area. Lodging was similar between the two trips as well, having the participants stay at a simple, centralized hotel within 1–2 h of the remote clinic sites. The GHOE groups were not differentiated in the survey to help preserve participant anonymity.

### Assessment tools to evaluate burnout

To evaluate burnout, we used the Copenhagen Burnout Inventory for students (CBI-S). The CBI-S has been validated to appropriately measure burnout in healthcare professionals and medical students across several languages [33, 34, 18]. The CBI-S was selected over the more popular Maslach burnout inventory (MBI) because it is a public domain questionnaire, making it free to use. It is also designed to be adapted to specific scenarios. We sought a shorter length survey to reduce attrition. The survey used was reduced to 9 questions, which were selected for the high Content Validity Ratio and adequate Confirmatory Factor Indices reported by the Campos et al. 2013 study [34]. CBI-S items were scored on a 1–5-point likert scale, all positively correlated to burnout. 4 questions focused on personal burnout (PB), 3 questions on studies related burnout (SRB), and 2 questions on colleagues related burnout (CRB). A score for each PB, SRB, and CRB was determined by the average response value within category and was then converted from the scale of 1–5 to 0-100. Magnitude of total score positively correlates with burnout. Severity of burnout was grouped as low (0-<50), moderate (50-<75) and high (75-<100), based off Kristensen’s description of the CBI [18]. PB questions focused on generalized exhaustion and fatigue, SRB questions focused on exhaustion and fatigue related to school attendance and studying, while CRB questions focused on exhaustion and fatigue related to working with teachers and peers. These questions are seen on the figures and tables tab.

### Statistical analysis

Data was analyzed using Generalized Linear Models that included the repeated measurement effect of each individual participant. The data was analyzed through three Models:

Model 1: estimation of PB, SRB or CRB means across time points.

Model 2: estimation of PB, SRB or CRB means across time points by GHOE/Control groups.

Model 3: estimation of PB, SRB or CRB means across time points by GHOE/Control groups while considering the effect of additional confounders (Medical School Year, Campus, Gender, Being Religious or Spiritual, and Number of Leisure hours a Week

The question “Have you been on a GHOE trip before” was excluded from the analysis because of conflicting responses in the pre-post-trip answers. Models were run using PROC MIXED in SAS/STAT v.9.4 (SAS Institute Inc., Cary NC). Normality assumptions were assessed through residual plots and Residual/Quantile plots. None of the models violated normality assumptions. All significant associations were declared at P ≤ 0.05, but exact probabilities are provided.

## Results

### Participation and sample

Demographics between the sample populations, GHOE Participants, and Control Participants on the initial survey (S1), 1 week post spring break (S2), and 10 weeks post spring break (S3) is compared in Table 1. 14, 14, and 7 responses were initiated for surveys 1, 2, and 3 respectively, but data was not collected due to lack of consent, academic probation, or an entirely incomplete form. There were no partially complete responses. While most responses could be longitudinally tracked, there was occasional user error including forgotten codename, incorrect entry, or missed survey, which are evident in the provided dataset.

**Table 1:** Descriptive statistics of sociodemographic variables in participating groups

	GHOE Participants			Control Participants		
	S1	S2	S3	S1	S2	S3
Number	n = 22	n = 20	n = 19	n = 70	n = 66	n = 50
Response Rate%	53.70%	48.80%	46.30%	27.80%	26.20%	19.80%
Male	10	10	10	27	25	15
	45.50%	50.00%	52.60%	38.60%	37.90%	30.00%
Female	12	10	9	43	41	35
	54.50%	50.00%	47.40%	61.40%	62.10%	70.00%
1st year	4	6	7	20	18	15
	18.20%	30.00%	36.80%	28.60%	27.30%	30.00%
2nd year	18	14	12	50	48	35
	81.80%	70.00%	63.20%	71.40%	72.70%	70.00%
Colorado	5	5	6	34	38	21
	22.70%	25.00%	31.60%	48.60%	57.60%	42.00%
Utah	17	15	13	36	28	29
	77.30%	75.00%	68.40%	51.40%	42.40%	58.00%
Age 22–23	0	1	1	2	2	2
	0.00%	5.00%	5.30%	2.80%	3.00%	4.00%
Age 24–29	20	18	16	62	60	42
	90.90%	90.00%	84.20%	88.60%	90.90%	84.00%
Age 30–40	2	1	2	6	4	6
	9.10%	5.00%	10.50%	8.60%	6.10%	12.00%
Religious/ Spiritual	13	14	11	42	47	32
	59.10%	70.00%	57.90%	60.00%	71.20%	64.00%
0–5 leisure hrs/wk	7	4	2	19	19	21
	31.80%	20.00%	10.50%	27.10%	28.80%	42.00%
6–10 leisure hrs/wk	8	8	10	30	28	14
	36.40%	40.00%	52.60%	42.90%	42.40%	28.00%
11–15 leisure hrs/wk	5	4	4	12	13	12
	22.70%	20.00%	21.10%	17.10%	19.70%	24.00%
> 15 leisure hrs/wk	2	4	3	9	6	3
	9.10%	20.00%	15.80%	12.90%	9.10%	6.00%

S1 = Initial Survey; S2 = Survey administered 1-week after spring break; S3 = Survey administered 10 weeks after spring break. Number and percentage of each response is reported.

### Burnout

The scores for PB, SRB, and CRB for all participants across the first survey are organized by cutoff values of 0–50, 50–75, and 75–100 to reflect relatively low, moderate, or high levels of burnout in Table 2. SRB scores were higher than other categories, with 89.1% of participants scoring 50 or higher.

**Table 2:** Participant distribution of burnout in the Initial survey

Measure	Mean score (Std)	Severity Cutoff Value	N (%)
PB, n = 92	59.24 (16.93)	Low (0-<50)	22 (23.91%)
		Moderate (50-<75)	47 (51.09%)
		High (75–100)	23(25.00%)
SRB, n = 92	69.57 (17.09)	Low (0-<50)	10 (10.87%)
		Moderate (50-<75)	36 (39.13%)
		High (75-<100)	46 (50.00%)
CRB, n = 92	42.66 (24.04)	Low (0-<50)	47 (51.09%)
		Moderate (50-<75)	32 (34.78%)
		High (75-<100)	13 (14.13%)
PB = personal burnout; SRB = studies related burnout; CRB = colleagues related burnout.			

The mean estimate for PB, SRB, and CRB scores for GP and CP across S1, S2, and S3 is compared in Table 3. Personal burnout, studies related burnout and colleagues related burnout did not significantly differ between timepoint 1 and timepoint 3. Personal Burnout estimates were significantly different between the GHOE and control only for model 2 for the last time point. Studies Related burnout estimates were significantly different between the GHOE and control for the last time point in both Model 2 and 3. This effect on PB and SRB was not observed at any other time point. Colleague Related Burnout displayed significant differences between the GHOE and control at the second and third time points. These differences were consistent and observed in Model 2 and 3.

**Table 3:** The impact of GHOE participation on burnout (PB, SRB, CRB)

	Survey 1 (S1)			Survey 2 (S2)			Survey 3 (S3)		
	Estimate	SE	P-value	Estimate	SE	P-value	Estimate	SE	P-value
**PB**									
Model 1									
Total Population	59.13	1.88	Not Applicable	57.28	1.95	Not Applicable	61.49	2.18	Not Applicable
Model 2									
GHOE	57.44	3.88	Not Significant	53.44	3.98	Not Significant	52.78	4.19	0.0161
Control	59.64	2.13		58.46	2.21		64.63	2.52	
Model 3									
GHOE	53.63	3.79	Not Significant	53.03	3.79	Not Significant	52.39	3.96	Not Significant
Control	57.45	2.15		56.19	2.31		60.04	2.57	
**SRB**									
Model 1									
Total Population	69.92	1.90	Not Applicable	65.59	1.97	Not Applicable	69.36	2.20	Not Applicable
Model 2									
GHOE	65.87	3.90	Not Significant	61.25	4.00	Not Significant	59.26	4.21	0.0056
Control	71.13	2.14		66.92	2.22		73.00	2.53	
Model 3									
GHOE	62.43	3.78	Not Significant	60.59	3.78	Not Significant	58.98	3.95	0.0402
Control	68.39	2.15		64.23	2.30		68.52	2.56	
**CRB**									
Model 1									
Total Population	42.58	2.54	Not Applicable	43.24	2.63	Not Applicable	42.10	2.94	Not Applicable
Model 2									
GHOE	38.69	5.19	Not Significant	30.63	5.32	0.0072	31.94	5.61	0.0357
Control	43.75	2.84		47.12	2.95		45.75	3.36	
Model 3									
GHOE	35.53	5.31	Not Significant	29.96	5.32	0.0051	30.66	5.55	0.0380
Control	42.93	3.02		46.83	3.24		44.23	3.61	

PB = personal burnout; SRB = studies related burnout; CRB = colleagues related burnout. P-values correspond to the comparison between the GHOE and control group by time point. S1 = Initial Survey; S2 = Survey administered 1-week after spring break; S3 = Survey administered 10 weeks after spring break. SE = Standard Error.

### Gender

The gender distribution among GHOE participants reflected the sample population more closely than control participants. Overall, more female participants responded to the surveys than male participants as seen in Table 1. Burnout results by gender are depicted in Table 4. Female participants had significantly higher burnout for PB, SRB (PB, P = 0.0044; SRB, P = 0.001; CRB, P = 0.6350), than male participants.

**Table 4:** Burnout scores reported by gender

Gender (n)	PB mean score (std dev)	SRB mean score (std dev)	CRB mean score (std dev)
Female (150)	63.1 (17.5)	72.8 (17.8)	43.4 (24.3)
Male (96)	53.6 (17.2)	61.3 (16.8)	41.9 (23.9)

PB = personal burnout; SRB = studies related burnout; CRB = colleagues related burnout.

### GHOE motivation factors

Motivation for attending the GHOE was ranked among trip participants. “Clinical experience” was the highest ranked motivator (48.4%) followed by “global travel experience” (41.9%), “make personal connections” (6.5%), and the lowest ranked factor was “improve curriculum vitae (CV)” (3.2%).

### Prior GHOE participation

On the initial survey, 16 out of 70 control participants (22.9%) reported prior GHOE participation. 12 out of 22 GHOE participants (54.5%) reported prior GHOE participation.

### Lifestyle factors

Higher leisure time was associated to lower PB, SRB (PB, P = 0.0001; SRB, P = 0.0001; CRB, P = 0.1837). Reported religious/spiritual (R/S) identity was evaluated throughout the study and cross analyzed with burnout scores. A smaller percentage of GHOE participants identified as R/S compared with control participants, Table 1. People who identified themselves as R/S consistently displayed lower burnout scores (PB, P = 0.0189; SRB, P = 0.0292; CRB, P = 0.0002).

### Alternative spring break options

Control participants reported on their planned spring break activity before and after spring break. Completed activities are reflected in S2, where 41 of 70 control participants reported their activity as rest/relaxation, 15 travel, 4 study, 4 research, 1 service, and 1 clinical experience. Several individuals with plans to study or travel on S1 retrospectively reported spending their spring break resting and relaxing on S2.

## Discussion

This study investigated burnout amongst medical students at RVUCOM. Burnout was measured among students attending a spring break global health trip compared with other spring break activities. Demographic and lifestyle factors such as the quantity of leisure time and spirituality were considered. To our knowledge, this is the first study to utilize a control group in objectively assessing Global Health Outreach Experiences’ impact as a curriculum component. The three-model approach used in this study allows for a comprehensive evaluation of the source of the effect. Model 1 being the simplest as it displays the pattern of burnout among all participants over time. The focused models (Model 2 and 3) provide context into each time point. Specifically, the change in burnout among GHOE participants compared to control participants is identified in Model 2, and whether confounders provide additional information in Model 3.

### Burnout

A significant reduction in personal burnout, studies related burnout, and colleagues related burnout was demonstrated at 10 weeks after spring break among GHOE participants compared with control participants in the model 2 analysis. No significant change in PB, SRB was found at 1-week after spring break, though reductions in CRB were significant at this time (Table 3). The increasing difference in burnout scores between GHOE and control participants at 10 weeks versus 1 week after spring break suggests that any beneficial effects of GHOEs may strengthen over time. These findings are supported in a prior study by Vu, et al., where feelings of perceived benefit (adaptability, communication, and cultural skills) described by medical students attending a medical mission trip, persisted years after participation in such experiences as medical students [35].

Burnout may fluctuate on a daily or weekly basis [36, 37]. Model 1 demonstrates there were not significant fluctuations across the population during the study period (Table 3). However, there was a steady increase in mean burnout estimates over time among the control participants. This finding is consistent with literature describing medical school’s effects of increasing burnout over time [4, 5].

In the initial survey, over 75% of individuals scored a mean value greater than or equal to 50 (potential score 0-100) on studies related burnout and personal burnout categories, reflecting high prevalence of burnout within these categories (Table 2). SRB was the highest, while CRB was the lowest scoring category across all surveys. Kristensen describes CBI categories as “the attribution of fatigue and exhaustion to specific domains or spheres in the person’s life” [18]. For example, colleagues-related burnout describes burnout symptoms that an individual attributes to interactions with colleagues. Considering SRB was the highest scoring category throughout all surveys, we can interpret the sample population attributes existing burnout to attending medical school, supporting medical school’s designation as the primary driver of burnout in students [6]). Reasoning why colleague related burnout was lower scoring, we can consider participants were in the didactic years of school, which primarily consists of self-study with little time coordinating on a team with others.

Model 3 analysis, which accounted for potential confounders, did not show significant association between GHOE attendance and PB at any time. SRB was reduced at 10 weeks post spring break among GHOE participants while CRB was reduced at both 1 week and 10 weeks post spring break among GHOE participants compared to control participants (Table 3). Potential confounders of PB and SRB included gender, religiousness/spirituality, and quantity of leisure time. CRB had different potential confounders, including R/S and campus. Model 3 findings are agreeable with model 2 in supporting GHOEs benefit on medical student burnout, specifically SRB and CRB.

There are mechanisms, consistent with known protective factors, that GHOE participation may reduce burnout. GHOEs could remind students of the reasons for practicing medicine through service and clinical exposure [38, 31]. The experience may help establish friendships with other attendees leading to improved social support [13, 4, 6]. Exposure to diverse conditions could improve resilience [12, 39]). A structured spring break may reduce maladaptive behaviors [40, 41]. The significantly reduced CRB in S2 and S3 among GHOE participants may support the mechanism of improved social network. There is likely a combination of factors impacting other aspects of burnout.

We recognize that other healthcare disciplines, such as physician assistant, nursing, or pharmacy, may also experience high levels of burnout [42]. Our literature review found far fewer burnout studies on these groups. Given the similar environment, educational content and associated stressors to medical school, we suspect that our findings regarding GHOEs may also translate to students and professionals of other healthcare disciplines.

### GHOE and control group comparison with additional associations

The most common alternative spring break activities completed by the control group participants were rest and relaxation (59%), followed by travel (21%). We considered rest and relaxation unlikely to create a similar experience to a GHOE due to the busy GHOE itinerary. The activity of travelling may share more features with GHOE attendance but is likely highly variable.

In the GHOE group, the strongest motivators for attending a trip were clinical experience followed by global travel. Observing that only one control participant completed clinical experience for an alternative spring break activity, while clinical experience was the highest ranked motivator for GHOE participation, we can consider there was a difference in initial mindset between groups.

Female participants had significantly higher levels of PB, SRB than males (Table 4). These findings are consistent with other assessments of medical students burnout [8, 37]. Female respondents were more heavily represented among control participants as seen in Table 1. Gender discrepancy between groups appears to be the most likely confounder discerned by the model 3 analysis.

Identifying as Religious/Spiritual was associated with decreased burnout, consistent with Wacholtz, 2013 study [43]. R/S may provide a framework to process stress [44]. R/S identity was similar between GHOE and control respondents.

Increased leisure time was significantly predictive of lower PB, and SRB. Leisure time was not associated with CRB. Limited leisure time is a known contributor to stress levels in graduate students [45] and residents [46]. A plurality of students reported 6–10 h leisure time weekly from both GHOE and control groups. Leisure time among medical students is a factor curriculum planners may have influence over and should also be considered in burnout prevention.

Utah students were more heavily represented among GHOE respondents compared to control. However, when comparing Utah and Colorado Campuses, there were no significant differences in burnout.

### Recommendations

Global health outreach opportunities have become commonplace in medical schools. In 2016, 140 schools offered third-year international electives [47]. The evidence presented in this study suggests GHOEs may be a beneficial offering for students. Medical schools should look at ways to further increase student involvement in GHOEs as one tool to reduce burnout.

The cost of attending GHOEs remains a significant barrier to include everyone interested. Schools can proactively plan fundraisers to reduce costs. Incorporating GHOE expenses into the estimated cost of tuition would allow financial coverage through subsidized loans. Trip availability and timing are other challenges. Spring break or summer break both offer ideal scheduling opportunities and have the benefit of integrating clinical exposure into students’ didactic years.

Some controversy exists regarding the ethics of Global Health Trips. Students may lack the preparation and skills necessary for clinical situations encountered [48]. Local needs are sometimes misread. Hosts may be overburdened [48]. Structural inequalities can be reinforced by taking away jobs and creating dependency [48]. If only volunteers benefit, such trips may become exploitative [48].

Ethically, the focus of GHOEs should be directed towards the beneficence, and non-maleficence of populations served [49]. We recommend these short-term experiences be nested within long-term sustainable programs. Expectations, support, and training should be technically adequate and culturally appropriate [29, 49]. The effectiveness of interventions should be regularly evaluated. GHOEs present an opportunity for mutually beneficial experiences.

### Limitations

The sample size of respondents who attended a spring break global health trip was small, n = 19–22. Response rate was 46.3-53.7% with 13.6% attrition from first to last survey for GHOE participants and 19.8-27.8% with 28.6% attrition from first to last survey for control-participants. Those experiencing high burnout may have been less motivated to complete the survey due to the associated exhaustion. On the other hand, burnout individuals may have been more interested in participating in a topic important to them. Self-selection bias may have occurred among trip participants, as inherent protective traits may relate to the desire to attend a GHOE. For instance, GHOE participants appeared to have a stronger intention to gain clinical experience as discussed. However, it is also possible burnout individuals were drawn to attending the trip to reinvigorate their studies. Lastly, the CBI-S scale is not as widely used as Maslach Burnout Inventory to measure burnout, limiting comparison to other studies.

## Conclusion

Participation in a weeklong global health outreach experience appeared to reduce burnout in 1st and 2nd year medical students, with effects improving over the span of several weeks. Medical Schools have a responsibility to address student burnout ensuing from their curriculum and culture. Offering global health experiences may be an effective tool with additional propensity to benefit underserved communities and enhance student education. Further research on experiential learning opportunities such as GHOEs to alleviate and protect against burnout syndromes may help provide students with approachable options to maintain their well-being. Improvements of burnout in the medical student population will in turn yield healthier physicians better able to care for patients.

## Electronic supplementary material

Below is the link to the electronic supplementary material.

Supplementary Material 1

## Data Availability

The datasets generated and/or analyzed during the current study are available in the Figshare repository, 10.6084/m9.figshare.22083446.v1.

## Abbreviations

GHOE: Global Health Outreach Experience
RVU: Rocky Vista University
RVUCOM: Rocky Vista University College of Osteopathic Medicine
OMS (I and II): Osteopathic Medical Student (first and second year)
IRB: Institutional Review Board
CBI(-S): Copenhagen Burnout Inventory (for students)
PB: Personal burnout
SRB: Studies related burnout
CRB: Colleagues related burnout
S1: Survey 1 (pre-spring break)
S2: Survey 2 (1 week after spring break)
S3: Survey 3 (10 weeks after spring break)
R/S: Religiousness/spirituality
