# Supplementary material for: The impact of global health outreach experiences on medical student burnout
Source: BMC Med Educ. 2023 Jun 26;23:476. doi: 10.1186/s12909-023-04451-6 (PMC10294532; doi:10.1186/s12909-023-04451-6)
Supplement: Supplementary file 1 — Supplementary Material 1 [file 12909_2023_4451_MOESM1_ESM.docx]

**The Impact of Global Health Outreach on Medical Student Burnout - Survey**

**Demographic/Background Questions**

1. Informed consent (I have read the information provided and I consent to participate in the study)
2. Are you in good academic standing? (mark yes or no)
3. Create a “codename” by entering the first 2 letters of your mother’s maiden name followed by the day of month of your mother’s birthday. Example Scott, April 13 Codename: SC13. If unknown, please pick a memorable four digit pin. This allows us to analyze your responses while keeping identifying information anonymous.
4. Year in medical school  (class of 2024, class of 2025)
5. Campus  (CO, UT)
6. Gender (male, female, transgender, non-binary, other)
7. Age
8. Do you consider yourself religious? (mark yes or no)
9. How many hours do you spend a week doing leisure activity? (0-5,6-10,11-15,15+)
10. Are you planning to attend an RVU Spring Break Global Trip in 2022? (mark yes or no)

11a. If Applicable (for students attending the trip): What was your primary motivation for applying/attending RVU Spring Break Global Trip?

*Rank from 1-4, 1 being most important, your response is anonymous*

-Global Travel Experience

-Improve Curriculum Vitae (CV)

-Make personal connections

-Clinical experience

11b. If Applicable (for students not attending the trip): What are you planning, or what did you do on your 2022 spring break?

*Pick one*

-studying

-clinical experience

-service

-rest/relaxation

-travel

12. Have you been on a global medicine outreach trip before? (mark yes or no)

13. I have a strong purpose that motivates me to study. (Strongly agree, agree, disagree, strongly disagree)

**Copenhagen Burnout Inventory Questions**

1=Never, 2=Rarely, 3=Sometimes, 4=Frequently, 5=Always

1. How often do you feel tired?
2. How often are you physically exhausted?
3. How often are you emotionally exhausted?
4. How often do you think: “I can’t take it anymore?”
5. Do you feel worn out at the end of the school day?
6. Are you exhausted in the morning at the thought of another day at school or studying?
7. Do you feel burn out because of your studies?
8. Does it drain your energy to work with teachers and peers?
9. Are you tired of working with teachers and peers?
